# Biogenetic space-guided synthesis of rearranged terpenoids

**DOI:** 10.1039/d3cc01009k

**Published:** 2023-05-10

**Authors:** Mykhaylo Alekseychuk, Philipp Heretsch

**Affiliations:** a Institute of Organic Chemistry, Leibniz Universität Hannover Schneiderberg 1B 30167 Hannover Germany philipp.heretsch@oci.uni-hannover.de

## Abstract

Natural product chemistry is constantly challenged by newly discovered, complex molecules. Elements of complexity arise from unprecedented frameworks, with a large amount of densely packed stereogenic centres and different functional groups along with a generally high oxidation state. As a prime example, rearranged triterpenoids possess all these elements. For their total synthesis, a limit of what is considered sensible in terms of steps and yield is frequently reached. As an alternative, semisynthetic approaches have gained a great amount of attention in recent years. In this featured article, we present our and others' contributions towards the development of efficient and economic syntheses of complex terpenoid natural products and elaborate on the underlying rationale of biogenetic space-guided synthetic analysis.

## Abstract 2

Biogenetic space-guided synthesis is a tool to design efficient chemical syntheses of complex terpenoids by evaluating their surrounding biogenetic space.

## Graphical abstract

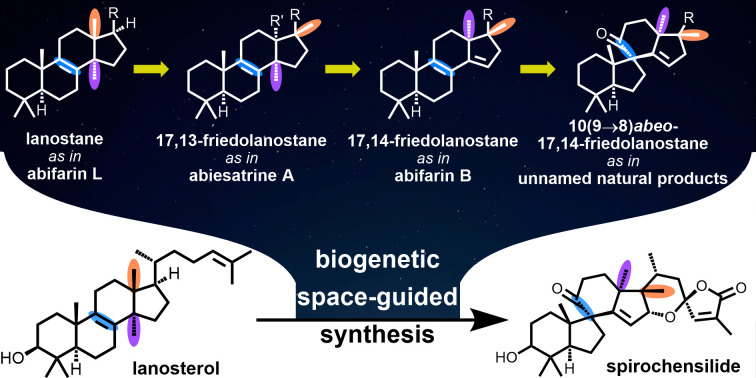

## Introduction

The synthesis of complex natural products has shaped the field of organic chemistry. In the last few decades, total synthesis, a discipline deemed mature by some, has started to evolve beyond what has been coined the “age of feasibility”. The focus has shifted from making a molecule at any cost to providing answers to the pressing demand for sustainable, facile and concise routes even to the most complex targets.^1^

Facilitating and streamlining access to the most complex natural products requires an understanding of nature's ways to biosynthesise these structures, *i.e.*, of their biogenesis. Biomimetic syntheses can then provide routes which frequently outrival conventional synthetic planning.^2^ In the absence of a plausible biogenesis proposal, this strategy is not accessible, though.

Our group and others have recently engaged in the synthesis of highly oxidised and rearranged triterpenoid natural products. In this review, we would like to highlight in several examples the rationale of biogenetic space-guided analysis, which we followed during our endeavours.

Thus, rigorously applying the following steps greatly helped to narrow down a synthetic problem. Accordingly, the chemical space around the natural product to be accessed is analysed. Co-isolated products as well as related natural products from close and more distant producing organisms are identified and then evaluated for a potential biosynthetic connection. Like assembling a puzzle, by putting these structural hints together, one can gather ideas on how nature may perform the relevant manipulations to transform a member of the standard repertoire of secondary metabolites into the natural product in question. In this analysis, unprecedented structural motifs, especially reconnections within the skeletal framework, are identified in a stepwise manner, with the intermediates being either already known isolated entities or those constituting anticipated natural products.

When this process of biogenetic space-guided analysis has provided a sensible biogenesis proposal, we set out to prove this hypothesis by chemically emulating the steps. If correct, replication of the key steps can be realised by chemical means (*i.e.*, biomimetically), employing the anticipated reactivity, and, thereby, supporting the biogenesis hypothesis. As a result of this process, not only the natural product, but also structurally related natural products *en route* to the final target can be obtained. Since the other intermediates encountered in the process all resemble the target structure to a certain degree, studying the structure–activity relationships (SARs) is another potential advantage and can provide answers to the question “which structural characteristics are necessary to provide a respective biological function?”

## Spirochensilide A and B

**Fig. 1:**
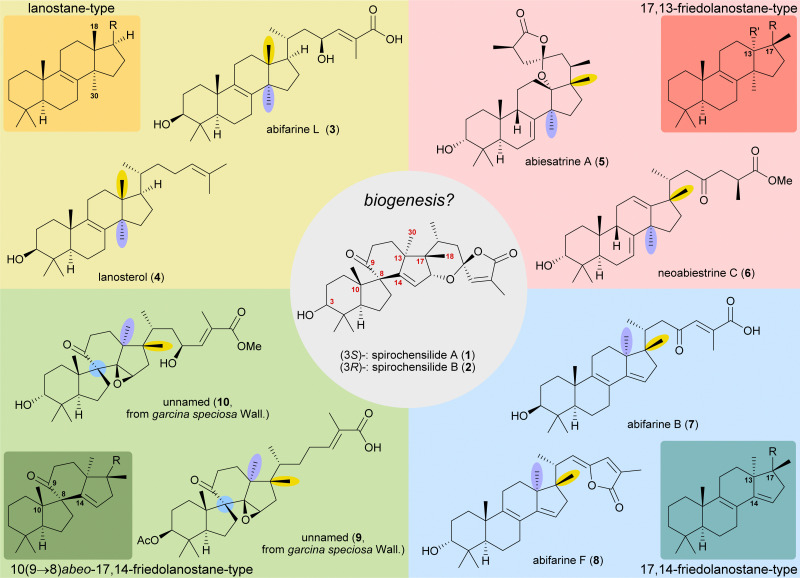
Natural products with related carbon frameworks in our biogenetic space-guided analysis towards spirochensilide A (1) and B (2).

The concept of biogenetic space-guided analysis is best demonstrated in the example of our synthesis of spirochensilide A (1) and B (2). These lanosterol-derived triterpenes were isolated by the group of Li and co-workers in 2015 from the Chinese fir *Abies chensiensis*.^3^ They feature a rearranged 10(9 → 8)abeo-17,14-friedolanostane-type carbon backbone with a spiro[4.5]decane and a 1,6-dioxaspiro[4.5]decene-motif (Fig. 1). Thus, the methyl groups 18 and 30 which reside at C13 and C14 in the lanostane skeleton have both moved by one carbon, to C14 and C17, respectively. Spirochensilide A (1) and spirochensilide B (2) differ in the stereoconfiguration at C3, with spirochensilide A (1) being the major isolated diastereomer.

The first total synthesis of spirochensilide A (1) was published in 2020 by Yang and co-workers^4^ with a Meinwald rearrangement^5^ and a Pauson–Khand reaction as key steps and proceeded in 27 linear steps from geranyl acetate. We first encountered the spirochensilides as potential targets for semisynthesis in 2019 and were instantly intrigued by their interesting structure, so we decided to take a deeper look into a plausible biosynthesis. During our investigation, we identified abifarines^6^ and abiesatrines,^7^ two classes of natural products containing 17,14-friedolanostane-type or 17,13-friedolanostane-type carbon backbones, respectively, isolated from other species of the *Abies* genus, *i.e.*, *Abies fargesii* for abifarines and *Abies georgei* Orr for abiesatrines (Fig. 1). Thus, in abifarine L (3) the standard lanostane framework is still unaffected, while abiesatrine A (5) and neoabiestrine C (6)^8^ are 17,13-friedolanostanes and abifarine B (7) and abifarine F (8) have a 17,14-friedolanostane carbon backbone, indicating that the shifts of the 18 and 30 methyl groups may occur in a stepwise and not concerted manner. When looking further into related natural products, we also noticed yet unnamed steroids 9 and 10, among others, displaying an 10(9 → 8)abeo-motif and, at the same time, migrated 18 and 30 methyl groups.^9^ We reasoned, the 17,14-friedolanostane carbon backbone may be a prerequisite for the later formation of the 10(9 → 8)abeo-motif, especially since no isolated natural products solely possessing the 10(9 → 8)abeo-motif, but without shifted methyl groups could be found. Extensive experiments by the isolators of the spirochensilides on lanostane-derived 8,9-epoxides to effect a Meinwald rearrangement, and thus forge the spiro-motif, failed, further supporting our assumption.^3^

**Scheme 1:**
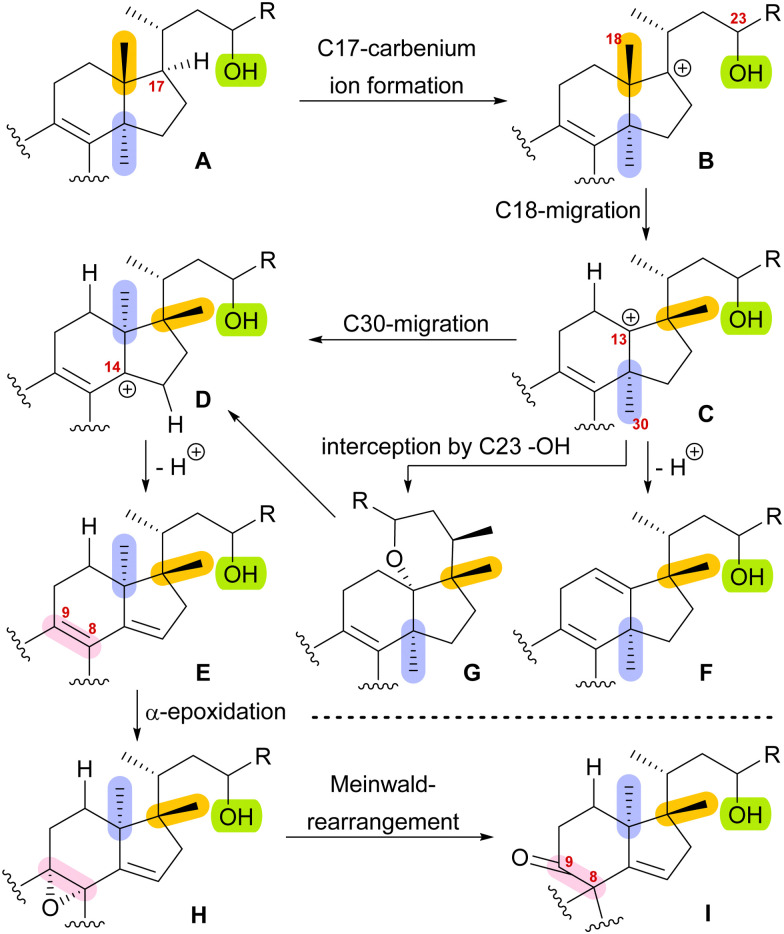
Proposed biosynthetic steps for the formation of 10(9 → 8)abeo-17,14-friedolanostanes based on biogenetic space-guided analysis.

With this hypothesis, we proposed a biosynthetic pathway for the 17,14-friedolanostane framework, as found in spirochensilide A (1) and B (2) (Scheme 1):^10^ Formation of a carbenium ion at C17 B is followed by a Wagner–Meerwein rearrangement of the 18-Me group from C13 to C17. The so-obtained carbenium ion at C13 C can then either undergo a second Wagner–Meerwein rearrangement of the 30-Me group from C14 to C13 to give cation D followed by proton elimination to yield diene E or directly lose a proton to give the 12,13-double bond in F, as in neoabiestrine C (6). Nucleophilic interception by the C23 OH-group can, as a third alternative, give tetrahydropyran G, as in abiesatrine A (5). This tetrahydropyran could, at a later point, also be reopened to regenerate the carbenium ion at C13 C and facilitate the 30-Me rearrangement. Diene E could then selectively be epoxidised from the α-face at the 8,9-double bond to give epoxide H, which could then rearrange in a Meinwald rearrangement to give the 10(9 → 8)abeo-17,14-friedolanostane framework I.

**Scheme 2:**
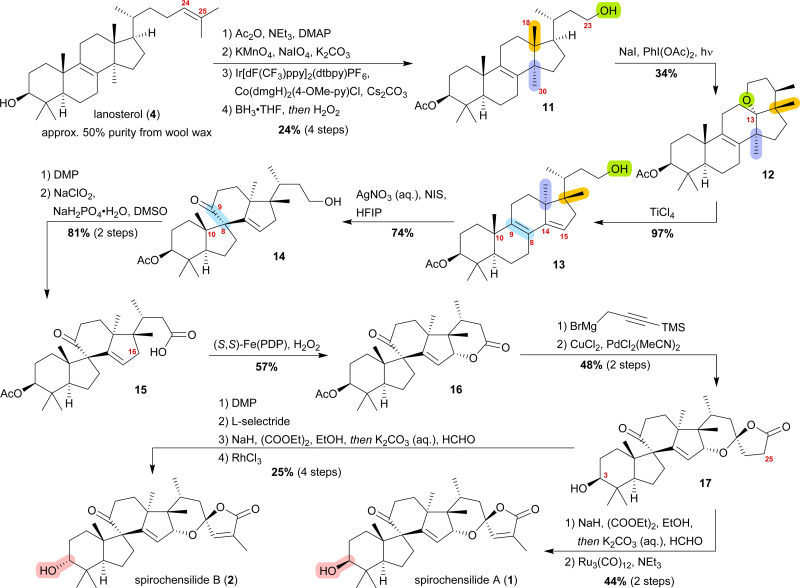
Semisyntheses of spirochensilide A (1) and B (2) by Heretsch and co-workers. ppy = 2-phenylpyridine, dtbpy = 4,4′-di-*t*butyl-2,2′-bipyridine, dmgH = dimethylglyoximato, (*S*,*S*)-PDP = (−)-2-(((*S*)-2-((*S*)-1-(pyridin-2-ylmethyl)pyrrolidin-2-yl)pyrrolidin-1-yl)methyl)pyridine.

**Scheme 3:**
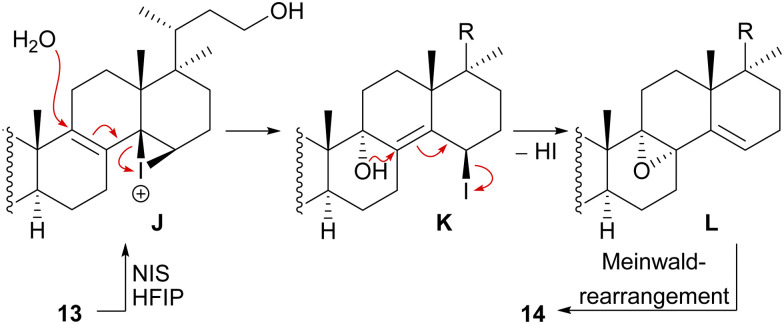
Mechanistic details of the iodonium-induced epoxidation of 13.

We decided to plan our synthesis around these rearrangements and employ lanosterol (4) as a starting material. To access the desired carbenium species in the unfunctionalised C17 position and without introducing a leaving group beforehand, we envisioned a radical process taking advantage of the distal hydroxyl moiety at C23, present, *e.g.*, in unnamed natural product 10, and use it for an H-atom transfer (HAT) process. Thus, acetylation of the C3-alcohol and Lemieux–von Rudloff oxidative scission^11^ of the side chain gave the 24-carboxylic acid (structure not shown), which was consecutively subjected to a photocatalytic decarboxylative elimination^12^ to give the terminal olefin which was then converted into the desired C23-alcohol 11 in a hydroboration/oxidation reaction (Scheme 2). The use of NaI, PhI(OAc)_2_ and light from a 45W CFL lamp gave the corresponding alkoxy radical which underwent 1,5-HAT.^13^ A radical-polar crossover then furnished the carbenium ion, which induced the Wagner–Meerwein rearrangement of the 18-Me group. Interestingly, and as predicted as a third alternative (*vide supra*), the alcohol at C23 then intercepted the C13 carbenium ion and formed tetrahydropyran 12. This product was readily transformed into the desired 17,14-friedolanostane 13 by the addition of TiCl_4_. Using so-obtained diene 13, epoxidation attempts only gave the 14β,15-epoxide instead of the required 8α,9-epoxide. This reactivity could be exploited by the formation of a fleeting 14β,15-iodonium ion J employing *N*-iodosuccinimide (NIS), AgNO_3_ and H_2_O (Scheme 3). Under these conditions, presumably, an intramolecular S_N_2′ reaction gave the desired 8α,9-epoxide L which instantly rearranged to give the 10(9 → 8)abeo-motif. The choice of solvent (hexafluoroisopropanol, HFIP) was critical for this reaction, as all other solvents tested led mostly to decomposition or, and only when the C23-alcohol was protected, to very low yields (3–14%).

The primary alcohol at C23 of 10(9 → 8)abeo compound 14 was then oxidised to carboxylic acid 15 using Dess–Martin periodinane (DMP) followed by Pinnick oxidation. Lactone 16 was formed in the following step by allylic oxidation using the White–Chen catalyst.^14^ The 1,6-dioxaspiro[4.5]decene system was then installed in a two-step procedure. First, addition of silylated propargylmagnesium bromide to lactone 16 and simultaneous deprotection of the acetate at C3, followed by cyclisation of the hemiacetal using PdCl_2_(MeCN)_2_/CuCl_2_ to afford lactone 17, was carried out. To complete the synthetic access to spirochensilide A (1), introduction of an exomethylene group in the α-position of the lactone and subsequent double bond isomerisation were necessary. For spirochensilide B (2), before the last two steps were carried out, first, the stereoconfiguration of the C3 alcohol of 17 had to be inverted by oxidation and diastereoselective reduction.

The synthesis of spirochensilde A (1) was, thus, completed in 13 steps from lanosterol (4), which, in comparison to the total synthesis by Yang and co-workers (27 steps), highlights the main advantage of semisynthesis over total synthesis.^4,10^

A few months after we reported our synthesis, a second semisynthetic approach was published by the group of Deng.^15^ Following a similar synthetic approach, they arrived at the same conclusion, *i.e.*, the Wagner–Meerwein rearrangements most likely have to precede the Meinwald rearrangement to render the latter possible. As their synthesis still differs from our approach, we here present a second example of a biogenetic space-guided analysis.

**Scheme 4:**
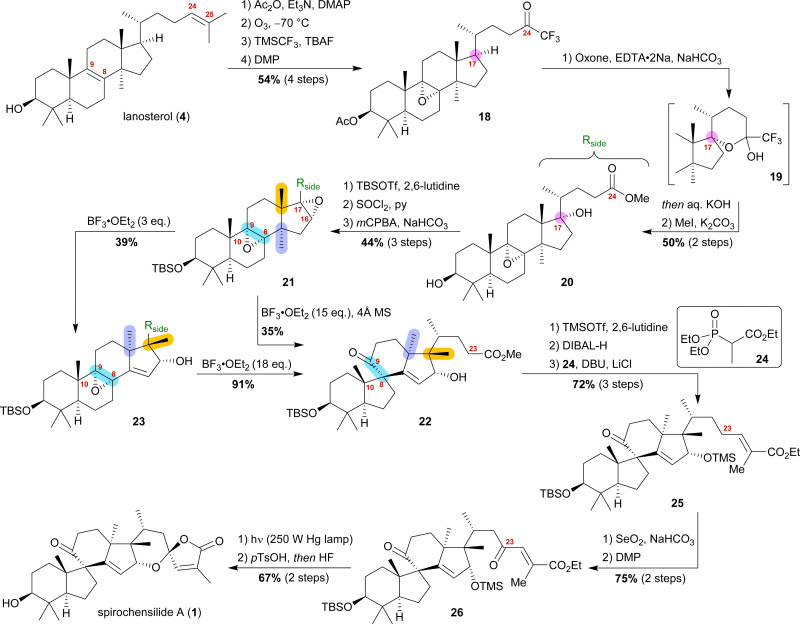
Semisynthesis of spirochensilide A (1) by Deng and co-workers.

Starting from lanosterol (4), the C3 alcohol was acetylated, and then ozonolysis at −78 °C led to the epoxidation of the 8,9-double bond while concomitantly cleaving the 24,25-double bond to provide an aldehyde, which was then converted to trifluoromethyl ketone 18 by reaction with TMSCF_3_ and subsequent oxidation with DMP (Scheme 4). The use of oxone and NaHCO_3_ initiated a dioxirane-mediated^15^ formation of hemiacetal 19, which was then hydrolysed to give C17 alcohol and C24 acid. Methylation in a consecutive step using MeI gave methyl ester 20. During this transformation, the acetate at C3 was removed and reprotection of the thus-obtained 3-hydroxyl group as a TBS-ether became necessary.

Elimination of the alcohol at C17 and epoxidation of the so-generated double bond then furnished 16α,17-epoxide 21. This epoxide was then rearranged to 10(9 → 8)abeo-17,14-friedolanostane 22 when treated with 15 eq. of BF_3_⋅OEt_2_. The authors further investigated this reaction and were able to perform the Wagner–Meerwein cascade without the consecutive Meinwald rearrangement by using only 3 eq. of BF_3_⋅OEt_2_. So-obtained epoxide 23 could then undergo Meinwald rearrangement when treated with excess BF_3_⋅OEt_2_. This competition experiment proved the order of rearrangements and further supported our results. Protection of the C16 alcohol of 10(9 → 8)abeo-17,14-friedolanostane 22 as a TMS-ether allowed for side chain extension *via* selective reduction of the methyl ester to the aldehyde using DIBALH and subsequent Horner–Wadsworth–Emmons reaction with propanoate 24 to give allylic ester 25. Riley oxidation at C23 and oxidation of the so-formed alcohol using DMP gave ketone 26. Finally, photoisomerisation of the 24,25-double bond was performed with subsequent TMS-deprotection under acidic conditions, leading to an intramolecular hemiacetalisation/lactonisation cascade and, thus, generating the dioxaspiro[4.5]decene motif. The addition of HF deprotected the C3 alcohol to yield spirochensilide A (1) in a total of 17 steps.^15^

Both syntheses show the power of biogenetic space-guided synthetic planning arriving at more step-efficient approaches (13 steps [Heretsch],^10^ 17 steps [Deng]^15^) in comparison to the total synthesis (27 steps [Yang]^4^).

## Strophasterol A and penicillitone

The synthesis of strophasterol A (27) is another example of biogenetic space-guided synthetic planning published by our group.^16^ In addition, it also furnished a versatile 14,15-secosteroid platform, applicable for the syntheses of several other 14,15-secosteroids as recently showcased in our syntheses of penicillitone (28)^17^ and asperfloketal A (54)^18^(*vide infra*).

Strophasterols A–D (27, 32–34) were first isolated from *Stropharia rugosoannulata* in 2012 by Kawagishi and co-workers,^19^ while strophasterols E (35) and F (36) were discovered later in 2019 by the group of Kikuchi from *Pleurotus eryngii*.^20^ Especially, strophasterol A (27) gained attention due to its mitigating effects on Alzheimer's disease.

**Fig. 2:**
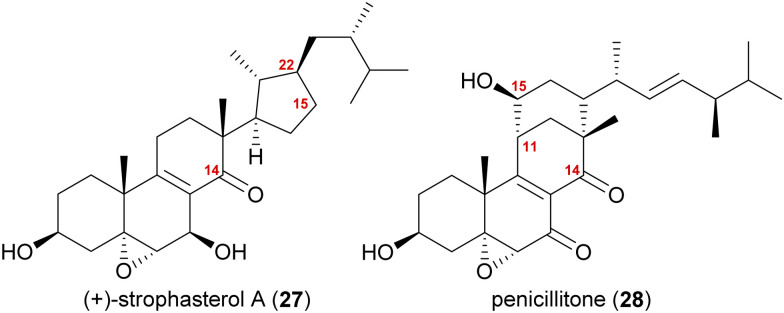
Structures of strophasterol A (27) and penicillitone (28).

**Scheme 5:**
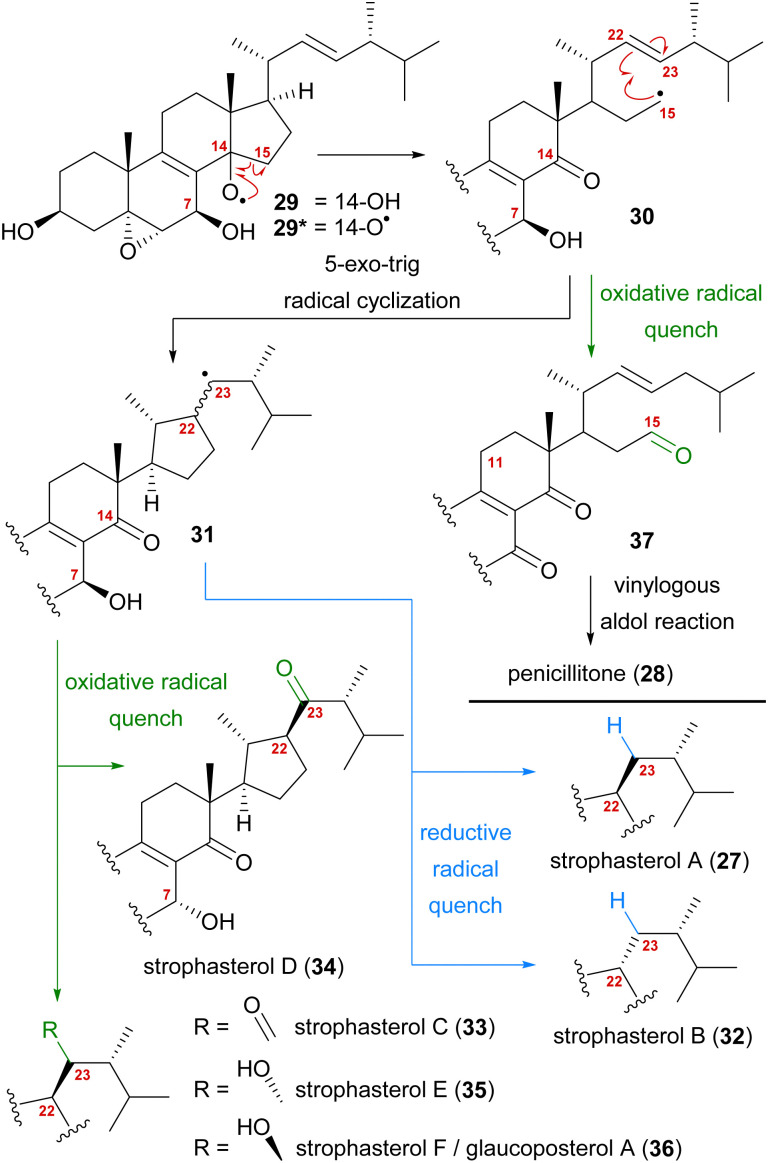
Initially proposed biosynthetic pathway towards strophasterols A–F (27, 32–36) and penicillitone (28) based on the 14,15-cleavage of co-isolated alcohol 29.

A first look at the connectivity of the carbon framework indicated a cleavage of the C14–C15-bond with C15 being reconnected to side chain C22 forming a five-membered ring (Fig. 2). In combination with the oxidation level of C23 (methylene or carbonyl), a radical cyclisation mechanism may be operative in the formation of this motif (Scheme 5). Analysis of the co-isolated natural products from *Stropharia rugosoannulata* furnished highly oxidised steroid 29 with a C14 alcohol and the A and B rings functionalised in a similar manner as in strophasterols.^19^ Building upon this information, we initially proposed 29 to be a biosynthetic precursor in which an alkoxy radical 29* would be formed from the C14 alcohol. This alkoxy radical could then initiate a β-scission of the C14,15-bond resulting in ketone 30 with a radical at C15. The latter would react in the 5-exo-trig radical cyclisation to furnish cyclopentane 31 with a radical at C23. A reductive quench of this radical would lead to either strophasterol A (27) or B (32), while an oxidative quench could lead to the formation of strophasterols C–F (33–36). Assuming an oxidative quench could also happen with the C15 radical 30, the formation of aldehyde 37 became a possibility. This aldehyde could react in an intramolecular vinylogous aldol reaction to furnish structurally related penicillitone (28). Penicillitone (28) was first isolated in 2014 by Wei and co-workers from *Penicillium purpurogenum* and was very recently synthesised for the first time by our group.^17,21^

**Scheme 6:**
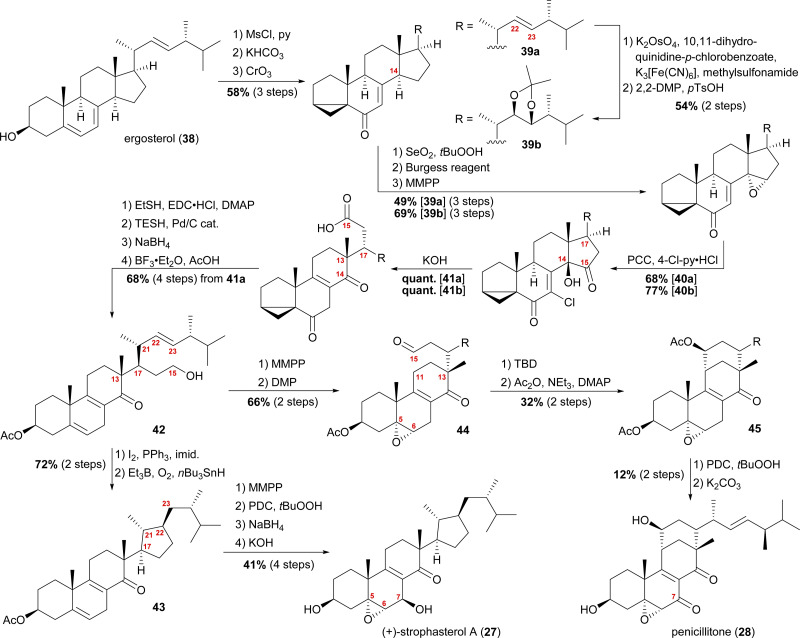
Semisyntheses of strophasterol A (27) and penicillitone (28) by Heretsch and co-workers. TBD = 1,5,7-triazabicyclo(4.4.0)dec-5-ene.

When we tried performing a radical-mediated 14,15 cleavage on a model substrate, however, we instead observed selective cleavage of the C13–C14-bond which was the starting point for our synthetic efforts towards dankasterone A (68) and B (69), as well as swinhoeisterol A (70) and periconiastone A (71), as will be discussed later. Although we chemically disproved the first step of our proposed radical biosynthetic pathway for this particular model system, a radical 14,15 cleavage may still be operative in a more closely related system and enzymatic environment, though. Our synthesis of strophasterol A commenced with the access to 14,15-steroid platform 41. Starting from ergosterol (38), masking the diene in the B ring was achieved by the formation of i-steroid 39a (Scheme 6).^22^ Towards the synthesis of asperfloketal A (*vide infra*), dihydroxylation and acetonide protection in the side chain was performed on the same i-steroid 39a. From there, the transformations towards the 14,15-seco platforms were identical for both, the 22,23-bishydroxy- and the Δ^22^-compounds, with slightly higher yields being obtained for the 22,23-diols, possibly due to the absence of an additional, potentially reactive, double bond. In the next step, i-steroids 39a/39b were oxidised at C14 using Riley conditions; the alcohol was then eliminated with the Burgess reagent and the resulting 14,15-double bond was reacted with magnesium monoperoxyphthalate (MMPP) to selectively give 14α,15-epoxides 40a/40b. Oxidation with pyridinium chlorochromate (PCC) and 4-chloropyridinium chloride gave α-chloro enones 41a/41b, which underwent 14,15-cleavage by treatment with KOH.^16^ Mechanistic insights into this two-step transformation can be found in our recent report on the synthesis of asperfloketal A (54).^18^ Acid 41a was then reduced to the C15-alcohol by first forming the corresponding thioester with ethanethiol and subsequent treatment with triethylsilane under Pd(0)-catalysis.^23^ Reduction of the C6-oxo moiety and unmasking of the i-steroid using AcOH/BF_3_⋅OEt_2_ gave alcohol 42. Conversion of the primary hydroxyl at C15 to the corresponding iodide and subsequent treatment with Et_3_B, *n*Bu_3_SnH and oxygen^24^ provided the intended cyclisation product 43 as a single diastereomer. With the core structure in place, epoxidation of the 5,6-double bond, allylic oxidation at C7 followed by reduction to the alcohol and, finally, deprotection of the acetate at C3 concluded the first synthesis of strophasterol A (27) in 18 steps from ergosterol (38). Against initial assumptions, the allylic oxidation at C7 did not take place in the bisallylic system 43, but was only successful after epoxidation of the 5,6-double bond.

Going back to alcohol 42, epoxidation of the 5,6-double bond using MMPP followed by oxidation of the C15 hydroxyl with DMP gave an aldehyde, which, under basic conditions (1,5,7-triazabicyclo(4.4.0)dec-5-ene, TBD), reacted *via* an intramolecular vinylogous aldol reaction with C11 to yield the penicillitone framework 45. When screening different bases, it turned out that only TBD was capable of facilitating the aldol reaction, presumably due to its ability to coordinate in the transition state.^25^ The synthesis of penicillitone (28) was then completed by acetate protection of so-generated C15 hydroxy steroid 45, oxidation at C7, and, finally global deprotection.^17^

**Scheme 7:**
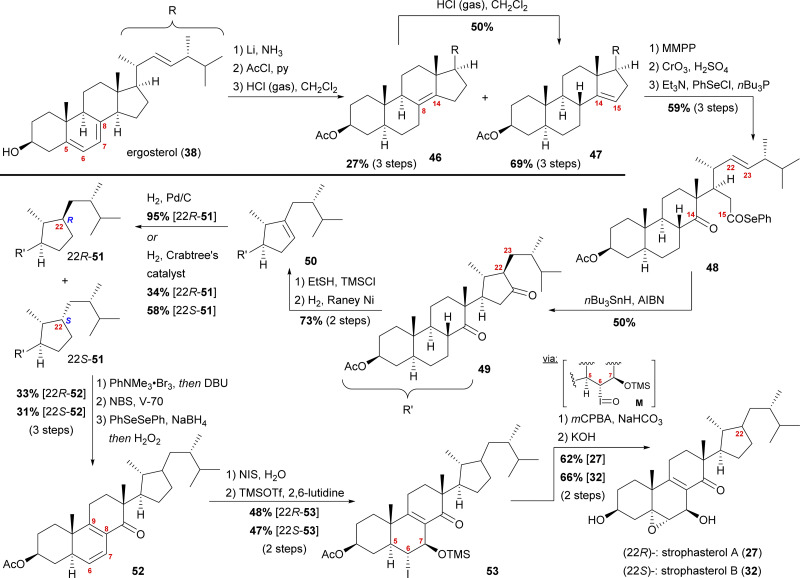
Semisyntheses of strophasterol A (27) and B (32) by Kuwahara and co-workers. V-70 = 2,2′-azobis(4-methoxy-2,4-dimethylvaleronitrile).

The group of Kuwahara later published a second semisynthetic approach towards strophasterol A (27) as well as strophasterol B (32) (Scheme 7).^26^ Following their initial studies, optimisation of their cyclisation step allowed them to synthesise strophasterols A–F (27, 32–36) in 2017–2020.^26–28^ Additionally, their work provided proof for the identity of glaucoposterol A and strophasterol F (36).^28^

Starting with ergosterol (38), the first goal was the cleavage of the C14–C15-bond. Therefore, the reduction of the 5,6-double bond under Birch conditions, protection of the C3 hydroxyl as an acetate, and isomerisation of the 7,8-double bond were performed. The latter reaction yielded both the 8,14-double bond isomer 46 as the minor product and the 14,15-double bond isomer 47 as the major product, with 46 being convertible into 47 under the same conditions. The 14,15-double bond was then cleaved by epoxidation and oxidative cleavage with CrO_3_ and H_2_SO_4_ giving the corresponding carboxylic acid, which was then reacted with PhSeCl to give selenoester 48. Using Boger's acyl radical formation method,^29^ selenoester 48 was treated with *n*Bu_3_SnH and AIBN to give cyclopentanone 49. Formation of thioenol ether using ethanethiol and TMSCl and subsequent desulphurisation with RANEY® nickel selectively gave cyclopentene 50, which was then hydrogenated to give selectively either 22*R*-cyclopentane 51 (Pd/C, H_2_) or a 1.7 : 1 mixture of 22*R*-51 : 22*S*-51-cyclopentanes when using Crabtree's catalyst. Both 22*R*-51 and 22*S*-51 could then be transformed to strophasterol A (27) or B (32) using analogous conditions. Thus, α-bromination at C8 with concomitant *in situ* dehydrobromination was performed giving the 8,9-double bond isomer. Allylic bromination at C7 was accomplished using *N*-bromosuccinimide (NBS) and 2,2′-azobis(4-methoxy-2,4-dimethylvaleronitrile) (V-70).^30^ Dehydrobromination turned out to be non-trivial, requiring first transformation of the bromide into a selenide and subsequently an oxidative elimination to give diene 52. This procedure could be performed as a one-pot sequence. The 6,7-double bond was then regioselectively transformed in a halohydrin reaction to furnish the 6α-iodo-7β-hydroxy moiety, which was TMS-protected in the following step, giving iodide 53. When treated with *m*-chloroperbenzoic acid (*m*CPBA), the 5α,6-epoxide was formed by first oxidation of the iodide to iodosyl M, elimination of the latter giving the 5,6-double bond, which, in turn, was epoxidised by *m*CPBA.^31^ Finally, global deprotection under basic conditions gave strophasterol A (27) or B (32), respectively, in a total of 17 steps each. The route of Kuwahara, thus, proves a similar radical cyclisation to be competent to forge the desired cyclopentyl motif. Towards this goal, a 14,15-secosteroid is traversed, potentially allowing access to penicillitone, and, thus pointing at a biogenetic relation.

## Asperfloketal A

**Fig. 3:**
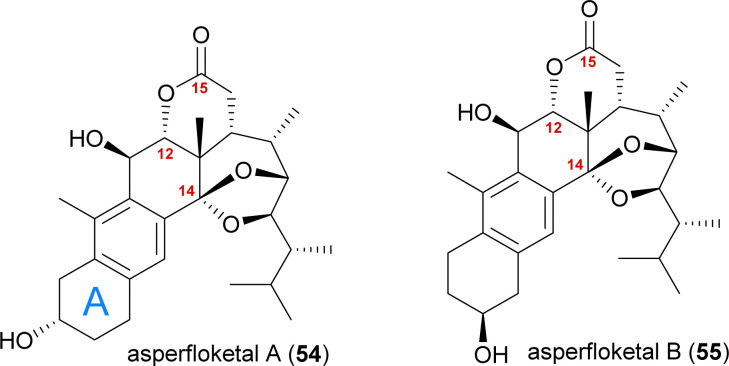
The asperfloketals A (54) and B (55).

The asperfloketals A (54) and B (55) are two anthrasteroids isolated in 2020 by Han, Xu, Lin and co-workers from *Aspergillus flocculosus* 16D-1, a fungus isolated from the marine sponge *Phakellia fusca*.^32^ These two regioisomeric natural products only differ in the position of the hydroxyl in the A ring, which prompted our interest in their biogenesis as well as in the biosynthetic formation of anthrasteroids itself (Fig. 3). Their complex rearranged skeletons include nine contiguous stereogenic centres (ten total), a ketal motif and a cleaved C14–C15-bond.

**Scheme 8:**
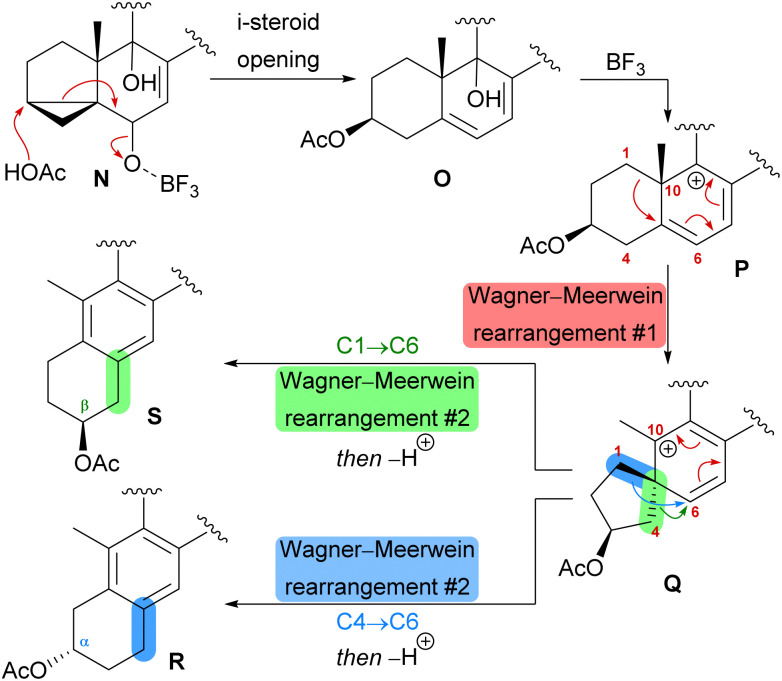
Proposed biomimetic anthrasteroid rearrangement mechanism.

When looking at all anthrasteroids isolated from natural sources until 2020, it became evident that only structures with a 3α-OH-group in the A ring had been reported, and no examples of anthrasteroids with a 2β-OH-group existed, with asperfloketal B (anthrasteroid numbering is different from steroid numbering, see Scheme 8) being the first reported.^33^ The isolators proposed a dehydration event taking place, which would be followed by rehydration to transform asperfloketal A (54) into asperfloketal B (55).^32^

The first synthetic anthrasteroids were obtained in 1954.^34^ As a proposed mechanism for their formation from steroid starting materials, two consecutive Wagner–Meerwein rearrangements giving first a 1(10 → 5)abeo-framework Q and then the 1(10 → 5),1(5 → 6)-diabeo-steroid R (Scheme 8, blue pathway) were discussed.^35^ Historically, rather harsh conditions were used for this rearrangement, often leading to low yields of the 3α-product (R), elimination of the alcohol and no 2β-product (S).^35,36^ Our initial intuition for an attempted chemical emulation of the anthrasteroid rearrangement required a substrate possessing a hydroxy group at C9 (as in O) to allow for milder conditions in the rearrangement. To account for the formation of both regioisomeric asperfloketals, we furthermore speculated that the C4–C5-bond in Q could migrate instead of the C1–C5-bond. Thus, in a mechanistic bifurcation event, both anthrasteroids could be formed from the same intermediate, which would also constitute a more straightforward biogenetic route. Further analysis of the asperfloketal framework revealed the C14–C15-bond to be cleaved oxidatively with the C14 oxo moiety forming the intramolecular ketal with a 22,23-diol in the process, while the carboxylic acid would connect to C12, and thus, yield the required lactone.

**Scheme 9:**
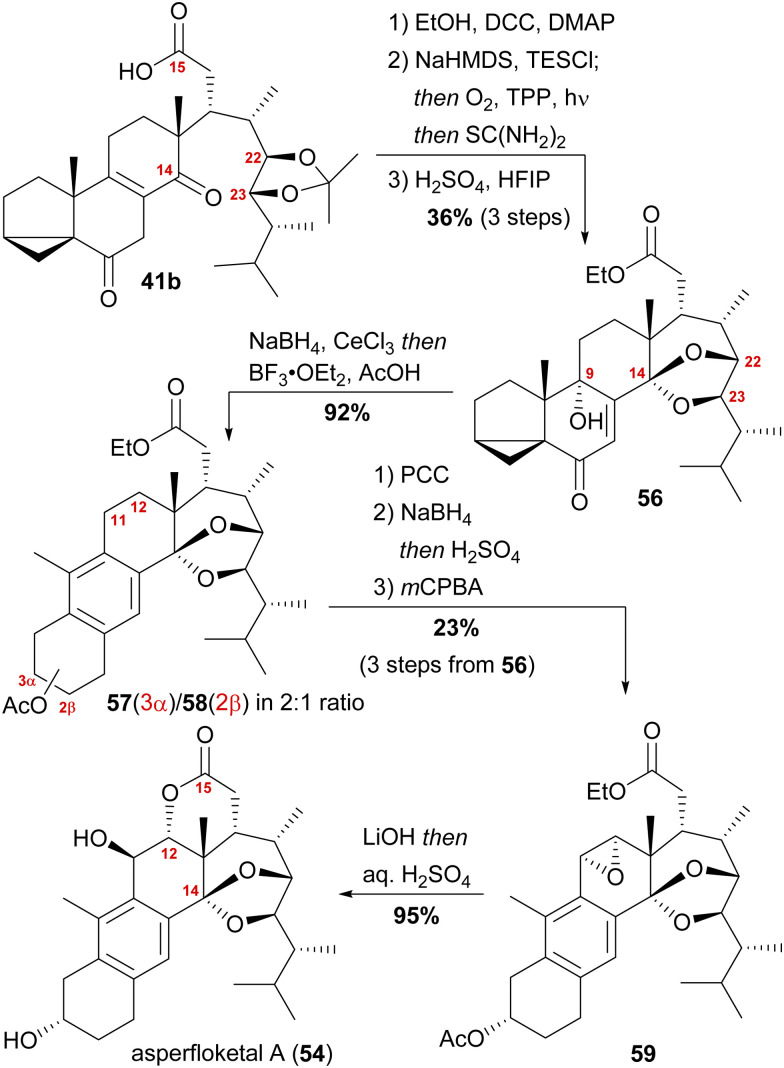
Semisynthesis of asperfloketal A (54) by Heretsch and co-workers.

**Scheme 10:**
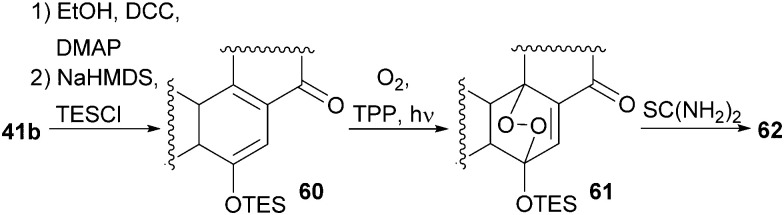
Mechanistic details of C9 oxidation of 41b.

With our previously synthesised 14,15-secosteroid platform, an additional dihydroxylation/acetonide protection step in position 22,23 (Scheme 6, 39 → 39b) was the starting point for the chemical emulation of the anthrasteroid rearrangement.^18^ Ketone 41b could, thus, be obtained in 10 steps. Then, the carboxylic acid was masked as an ethyl ester, and the hydroxy moiety at C9 was introduced by formation of silyl enol ether 60, [4+2]-addition of oxygen to the so-obtained diene and cleavage of the endoperoxide 61 (Scheme 9 and Scheme 10).

Finally, intramolecular transketalisation was accomplished by using aq. H_2_SO_4_ in HFIP. Enone 56 was then reduced to the allylic alcohol and treated with BF_3_⋅OEt_2_ in acetic acid to give the rearranged anthrasteroids 57/58, by first unmasking the 5,6-double bond (Scheme 8, N → O) and then generating the carbenium ion at C9 (Scheme 8, P). To our delight, this reaction sequence gave two products in excellent yields, which after extensive 2D NMR analysis turned out to be the regioisomeric anthrasteroid rearrangement products in a 2 : 1 (57 : 58) ratio. From there, oxidation at the benzylic C11, reduction and elimination and, finally, epoxidation, selectively gave 11α,12-epoxide 59. Global deprotection then yielded asperfloketal A (54) in 18 steps from ergosterol (38). We assume the final cyclisation of the lactone to proceed by first opening of the epoxide through a benzylic S_N_2 reaction with hydroxide to give the 11β,12α-diol, followed by transesterification during acidic work-up. When using aq. HCl instead of aq. H_2_SO_4_ for the acidic work-up, we could also observe the formation of 11β-chloro asperfloketal A (not shown) as a side-product, supporting this mechanistic rationale.

## Dankasterone A and B, periconiastone A and swinhoeisterol A

The first natural products of this group to be isolated were dankasterone A (68) in 1999 from the *Halichondria* sponge-derived fungus *Gymnascella dankaliensis* by Amagata, Minoura, Numata and co-workers,^37^ followed by dankasterone B (69) in 2007 from the same fungus and by the same group.^38^ Seven years later, Riccio, Bifulco, Gerwick, Zhang and co-workers were able to isolate swinhoeisterols A (70) and B (not shown) from the marine sponge *Theonella swinhoei*.^39^ More recently, periconiastone A (71) was isolated in 2019 by Liu, Hu, Zhang and co-workers from the endophytic fungus *Periconia sp.* TJ403-rc01.^40^

**Scheme 11:**
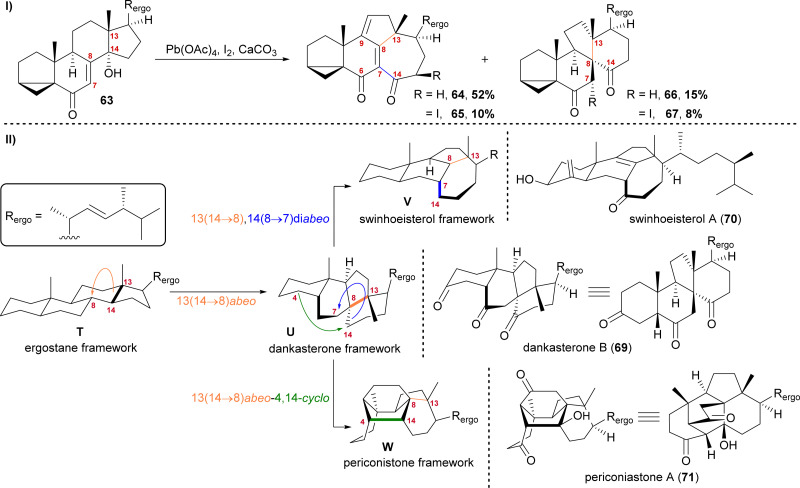
I: Alkoxy radical initiated framework rearrangement. II: Biogenetic space-guided analysis of dankasterone B (69), swinhoeisterol A (70) and periconiastone A (71).

We first started investigating this group of natural products when looking for a radical 14,15-scission approach towards the synthesis of strophasterol A (27)^16^ using a hydroxy group at C14 63 to initiate the scission. When generating the corresponding alkoxy radical, a mixture of ketone 66/67 and dienone 64/65 was formed (Scheme 11-I).^41,42^ While 66 and 67 possessed the 13(14 → 8)abeo-framework U, as it can be found in dankasterone A (68) and B (69), 64 and 65 possessed the 13(14 → 8),14(8 → 7)diabeo-framework V which corresponds to swinhoeisterol A (70) (Scheme 11-II). When investigating the mechanism of formation of both products, we realised the structural connection between the dankasterones and the swinhoeisterols, a suspicion further deepened by the geographical proximity of their respective places of isolation, *i.e.*, the Sea of Japan and the South China Sea, respectively.^37–39^ Thus, the rearrangement might proceed through the dankasterone framework U as an intermediate. As an integral part of the transformation of the dankasterone into the swinhoeisterol framework V, we assume a Dowd–Beckwith rearrangement.^43^ The proposed radical mechanism can be found in our original report. While this project was already in progress, the structure of periconiastone A (71) was published. An intramolecular aldol reaction was proposed for the formation of the C4–C14-bond to give the 13(14 → 8)abeo-4,14-cyclo framework W, directly from dankasterone B (69).

**Scheme 12:**
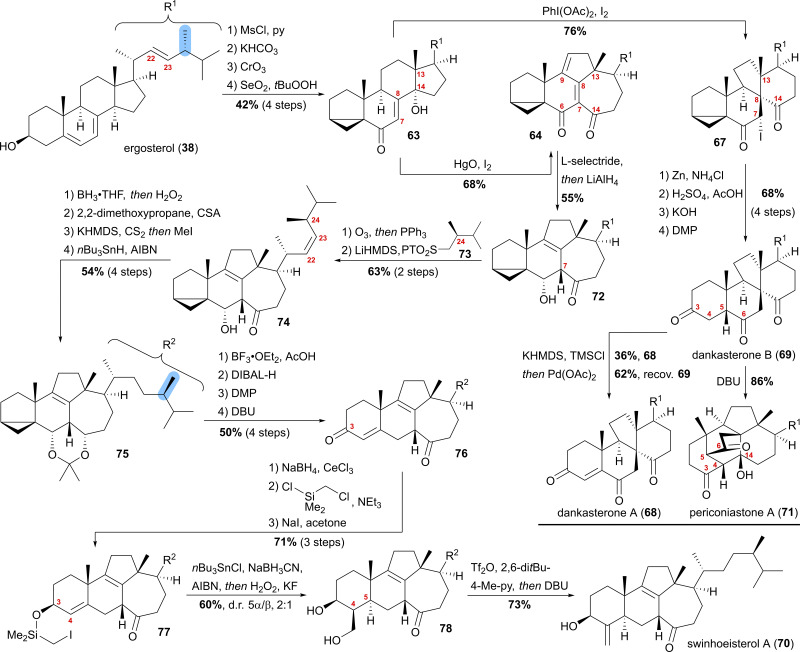
Semisyntheses of dankasterone A (68) and B (69), swinhoeisterol A (70) and periconiastone A (71) by Heretsch and co-workers.

Our synthesis started from ergosterol (38), which, after i-steroid formation and allylic oxidation, gave previously reported alcohol 63 (Scheme 12). Upon subjecting this alcohol to (diacetoxyiodo)benzene and iodine treatment, selective formation of α-iodide 67 possessing the dankasterone skeleton was observed in good yields. Deiodination with zinc, opening of the i-steroid with H_2_SO_4_ in acetic acid, followed by deprotection of the partially acetate-protected mixture led, after oxidation of the 3-hydroxy moiety with DMP, to dankasterone B (69) in only 9 steps from ergosterol (38). To transform dankasterone B (69) into dankasterone A (68), Saegusa–Ito oxidation of the corresponding silyl enol ether was performed, giving a separable mixture of dankasterone A (68) and reisolated dankasterone B (69).

To put the biosynthesis hypothesis of periconiastone A (71) to a test, we then investigated the propensity of 69 to undergo an intramolecular aldol addition between C4 and C14. Thus, diazabicycloundecene (DBU) was employed as a base, which indeed gave periconiastone A (71) as a single diastereomer and in an excellent yield. When trying to selectively synthesise the swinhoeisterol framework from alcohol 63, instead of (diacetoxyiodo)benzene, mercuric oxide and iodine were employed to give dienone 64 with complete selectivity and good yields. To reduce undesired reactivity when introducing the required side chain with a (24*R*)-configured methyl group, first, the diene in 64 was reduced in a 1,6-fashion with l-selectride. Subsequently, and in the same pot, LiAlH_4_ was used to reduce the C6 oxo-moiety to give 6α-alcohol 72 with an 8,9-double bond. With compound 72 in hand, regioselective ozonolysis of the 22,23-double bond was performed, followed by *Z*-selective Julia–Kocienski olefination with sulfone 73, which was prepared in six-steps from (*R*)-Roche ester.^44^ To circumvent epimerisation at C24, a reactivity observed when hydrogenation of 74 was attempted with Pd or Pt and H_2_, hydroboration/oxidation was performed to yield mostly the 23-hydroxy-isomer and no detectable epimerisation at C24, while concomitantly the oxo-moiety at C14 was reduced. Protection of the 6α,14α-diol as an acetonide followed by Barton–McCombie deoxygenation of the 23-hydroxy moiety then gave acetonide 75. When treated with BF_3_⋅OEt_2_ in acetic acid, the acetonide was deprotected, while at the same time, opening of the i-steroid gave the C3 acetate and a 5,6-double bond. Non-basic deprotection of this acetate with DIBALH, global oxidation of the alcohols to the corresponding ketones, and finally, DBU mediated 5,6-double bond isomerisation gave enone 76. To introduce the missing exomethylene group at C4 and to generate the correct stereoconfiguration at C5, the enone was reduced under Luche conditions giving the 3β-allylic alcohol, which was silylated using (chloromethyl)chlorodimethylsilane (product not shown). The Finkelstein reaction yielded iodide 77, which in the presence of AIBN, *n*Bu_3_SnCl and NaBH_3_CN underwent a Nishiyama–Stork radical cyclisation^45^ to give a dimethyl oxasilolane. Subjecting the latter to Tamao's conditions^46^ gave diol 78. Elimination of the primary alcohol by triflation, followed by the addition of DBU, then completed the first synthesis of swinhoeisterol A (70) in 21 steps from ergosterol (38).

These divergent syntheses of dankasterones A (68) and B (69), periconiastone A (71) and swinhoeisterol A (70) are valuable examples of a biosynthetic proposal to connect seemingly unrelated natural products and discovering and exploring novel reactivity.^41^ Only by careful analysis of the migration events needed to (formally) convert ergosterol into swinhoeisterol, the intermediacy of the dankasterone and periconiastone natural products was realised. The former served as a mechanistic bifurcation point and helped to emulate chemically the reaction cascade to arrive at the swinhoeisterol class of natural products. The effortlessness by which these rearrangements could be realised under radical conditions further points to a possible radical nature of these transformations during their biogenesis.

## Pleurocin A/matsutakone and pleurocin B

Two representatives of the group of 11(9 → 7)abeo steroids are the natural products pleurocin A/matsutakone (79) and pleurocin B (80), with pleurocin B (80) being the 22,23-dihydro-derivative of the former. The two names pleurocin A and matsutakone (79), respectively, can be traced back to the almost simultaneous isolation reports by the groups of Feng and Liu from *Tricholoma matsutake*^47^ and by Tanaka and co-workers from *Pleurotus eryngii*^48^ in 2017. Pleurocin B (80) was co-isolated together with pleurocin A (79) from *Pleurotus eryngii*.

While both groups independently put forward biosynthetic proposals of polar mechanisms for the C9–C11-bond cleavage and the C7–C11-bond formation reaction, a closer look at the structure and functional group distribution convinced us that a 6-*endo*-trig radical cyclisation, initiated by a C11 based radical in a molecule such as 89 (*vide infra*), followed by trapping of oxygen by a C8-centred radical could better explain the formation of the natural products from a similar biosynthetic precursor. To test this hypothesis, the synthesis of a 9,11-secosteroid platform from ergosterol (38) was attempted first.^49^

**Scheme 13:**
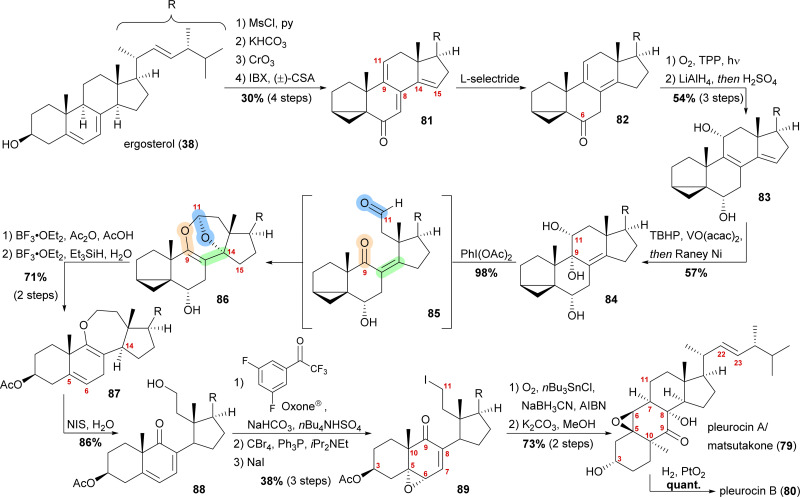
Semisyntheses of pleurocin A/matsutakone (79) and pleurocin B (80) by Heretsch and co-workers.

By forming the i-steroid 39a from ergosterol and treating the latter with 2-iodoxybenzoic acid (IBX) and camphorsulfonic acid (CSA), tetraene 81 was generated (Scheme 13).^50^ While studying this reaction, it became clear that first, the 14,15-double bond was introduced, followed by the 9,11-double bond, as no conditions were found to selectively generate the 9,11-double bond. Conjugate reduction with l-selectride gave 1,3-cyclohexadiene 82 which readily reacted *via* an [4+2] addition with singlet oxygen in the presence of TPP to provide the corresponding endoperoxide (not shown). Treatment of the latter with LiAlH_4_ reductively cleaved the endoperoxide while also reducing the C6 oxo moiety, resulting in 11α,14α-diol. Under strongly acidic work-up conditions, elimination of the C14 hydroxyl moiety could be observed, resulting in the formation of diene 83. Regio- and diastereoselective epoxidation of the 8,9-double bond, followed by immediate vinylogous reductive opening by addition of RANEY® nickel, gave the desired 9α,11α-diol 84, leaving the 22,23-double bond unscathed in the process. The cleavage of the C9–C11-bond was then achieved by addition of (diacetoxyiodo)benzene, presumably giving aldehyde 85, which then underwent an intramolecular dioxa-[4+2]-cycloaddition to form oxepane acetal 86. Treatment of the latter with BF_3_⋅OEt_2_ and acetic anhydride in acetic acid unmasked the i-steroid and also initiated β-elimination of H15, forming the 14,15-double bond while opening the tetrahydrofuran to the corresponding lactol acetate (not shown). Ionic reduction of C11 with Et_3_SiH and BF_3_⋅OEt_2_ as well as reduction of the extended enolate 14,15-double bond under the same conditions then provided enol ether 87. Hydrolysis of the enol ether was achieved by subjecting the molecule to aqueous NIS, which presumably first gave a 9,10-iodonium ion, which was then hydrolysed to yield an α-iodo ketone. Elimination of this α-iodide introduced the 7,8-double bond in 88. Regio- and stereoselective epoxidation of the 5,6-double bond was then achieved by *in situ* generated dioxirane from 2,2,2,3′,5′-pentafluoroacetophenone.^51^ The C11 hydroxy moiety was converted stepwise into iodide 89 through the intermediacy of a bromide. The stepwise conversion was necessary as a direct conversion of the hydroxy moiety to the iodide 89 under Appel-type conditions led to the deoxygenation of the epoxide. To complete the synthesis, the required C11-centered radical was formed by using Nakamura's conditions (1.5 eq. of O_2_, NaBH_3_CN as a stoichiometric reductant and catalytic amounts of AIBN and *n*Bu_3_SnCl).^52^ This oxidative radical cyclisation was followed by deacetylation to give pleurocin A/matsutakone (79) as a single diastereomer. Hydrogenation of the 22,23-double bond of the latter also provided pleurocin B (80) in quantitative yield.^49^

## Pinnigorgiols B and E

Pinnigorgiol B (90) and pinnigorgiol E (91), the 11-acetyl derivative of the former, were both isolated in 2016 by Sung and co-workers from a Taiwanese gorgonian coral *Pinnigorgia* species.^53^ In addition, pinnisterol E (92) was isolated, which is assumed to be the biosynthetic precursor, and thus, became key to devising a synthetic access (*vide infra*).

Pinnigorgiol B (90) has a tricyclo[5.2.1.1]decane framework and a γ-diketone moiety structurally resembling related aplysiasecosterol A (not shown) which was isolated in 2015^54^ and synthesised in 2018 by the group of Li.^55^ Pinnigorgiol B is both a 9,11-seco and a 5(6 → 7),6(5 → 10)diabeo steroid. We chose to highlight the approach by Gui and co-workers, as it is an impressive example of a semisynthesis taking into consideration the (hypothetical) biogenesis.^56^

**Scheme 14:**
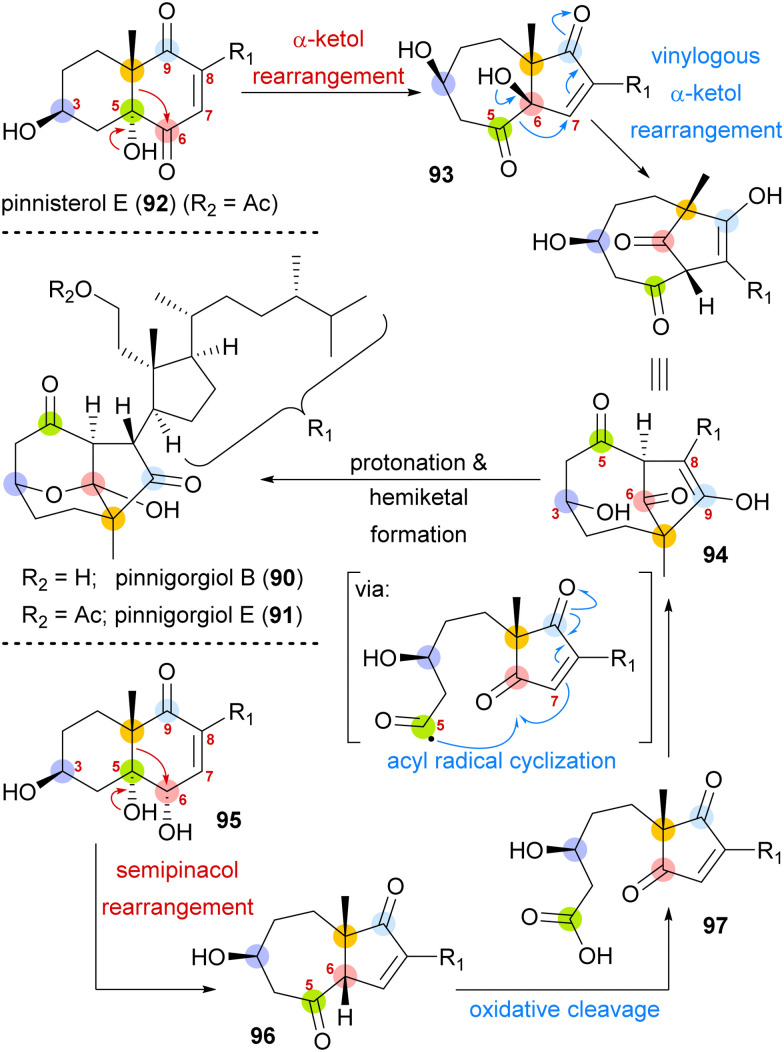
Proposed biosynthetic pathway towards pinnigorgiol B (90) and E (91) from pinnisterol E (92) and reactivity-based synthesis plan by Gui and co-workers.

A biosynthetic proposal by Kigoshi and Kita envisioned an α-ketol rearrangement taking place in co-isolated pinnisterol E (92) to yield α-hydroxy ketone 93, which then could react in a second, this time vinylogous α-ketol rearrangement, to yield the key diketone 94 (Scheme 14). As diketone 94 should be transformed into the natural product under acidic catalysis, Gui defined 94 as a key intermediate to their approach. When analysing α-hydroxy ketone 93, they found reports of structurally similar systems undergoing an undesired, facile C10 migration from C6 to C5. To circumvent this problem, they opted to develop a stepwise approach: starting with *syn*-diol 95 and accessing diketone 96 in a semipinacol rearrangement.^57^ Subsequent oxidative cleavage of the C5–C6-bond should give acid 97, which then could be transformed to the same key diketone 94 through an acyl radical cyclisation.

**Scheme 15:**
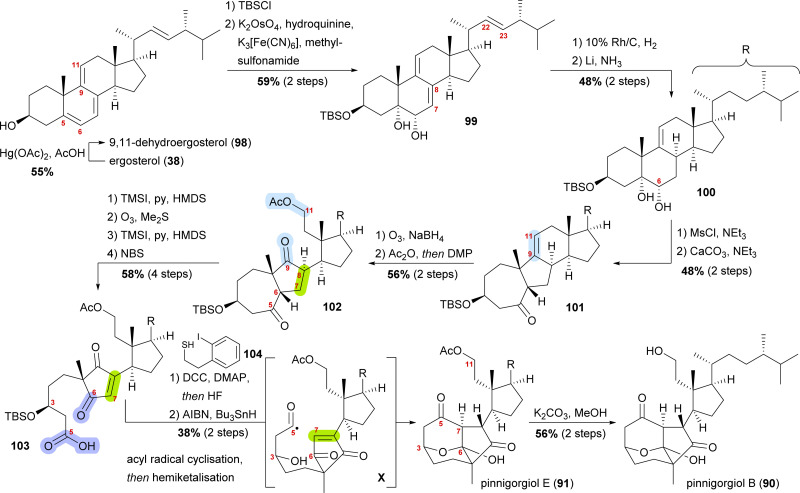
Semisyntheses of pinnigorgiol B (87) and E (88) by Gui and co-workers.

Starting from ergosterol (38), Treibs conditions were used to introduce a 9,11-double bond (98, Scheme 15).^58^ Next, the C3 hydroxyl was protected as a TBS ether and the 5,6-double bond was regio- and diastereoselectively dihydroxylated to furnish diol 99. Hydrogenation over rhodium on carbon selectively reduced the 22,23-double bond, while Birch reduction could then be employed to selectively reduce the 7,8-double bond to obtain 100, leaving only the 9,11-double bond unchanged. Extensive studies were performed to arrive at this desired outcome. Mesylation of the C6 hydroxyl allowed for subsequent semipinacol rearrangement and gave ketone 101.^59^ Ozonolysis of the 9,11-double bond followed by reductive work-up gave the corresponding diol, which was then selectively acetylated at C11 and then oxidised at C9 to provide diketone 102. Regioselective formation of silyl enol ether^60^ from the C5 oxo moiety, followed by ozonolysis of the enol ether subsequently gave acid. The 7,8-double bond was then introduced by the regioselective formation of silyl enol ether from the C6 oxo moiety and subsequent reaction with *N*-bromosuccinimide. Acid 103 was transformed into the corresponding thioester with thiol 104, and the TBS ether was removed. Treatment with AIBN and Bu_3_SnH generated the desired acyl radical X,^61^ which, in an 8-*exo*-trig cyclisation followed by direct hemiketalisation, gave pinnigorgiol E (91). Hydrolysis of acetate then gave pinnigorgiol B (90), completing a 16-step synthesis from ergosterol (38).

## Conclusions

In this review, we outlined the advantages of rigorously following biogenetic space-guided analysis to access complex terpenoid natural products in a semisynthetic fashion. Thus, by evaluating the structural space of co-isolated and related natural products, insights into the biosynthetic transformations and their order can be gained. With a general scheme for the possible biogenesis of the natural product in question, a chemical synthesis can then be planned and reduced to practice, thus elucidating innate reactivity, generating hypothesised intermediates and accessing anticipated natural products. This approach has, in our and other hands, helped to streamline synthetic routes, allowed access to additional related natural products *en route* and most importantly, provided chemical support for the biogenetic hypotheses. During the case studies presented in this review, we have encountered on several occasions that polar reactions were not sufficient in accessing the desired natural products and their intermediates, but rather an intricate interplay of radical and polar reactivity was necessary to successfully reduce a synthetic plan to practice. Especially the further development of radical and radical-polar crossover logic is a current pursuit of our group.

## Author contributions

Mykhaylo Alekseychuk wrote the original draft. Philipp Heretsch supervised the writing process and edited the manuscript.

## Conflicts of interest

The authors declare no competing financial interest.

## Supplementary Material
